# Nanotechnological prospective for enhancing the antibacterial activity of mupirocin and cinnamon essential oil: a combination therapy

**DOI:** 10.3389/fphar.2024.1468374

**Published:** 2024-11-11

**Authors:** Bandar Aldhubiab, Rashed M. Almuqbil, Tamer M. Shehata, Wafaa E. Soliman, Heba S. Elsewedy

**Affiliations:** ^1^ Department of Pharmaceutical Sciences, College of Clinical Pharmacy, King Faisal University, Al Hofuf, Saudi Arabia; ^2^ Department of Pharmaceutics, College of Pharmacy, Zagazig University, Zagazig, Egypt; ^3^ Department of Biomedical sciences, College of Clinical Pharmacy, King Faisal University, Al Hofuf, Saudi Arabia; ^4^ Department of Microbiology and Immunology, Faculty of Pharmacy, Delta University for Science and Technology, Mansoura, Egypt; ^5^ Department of Pharmaceutical Sciences, College of Pharmacy, AlMaarefa University, Riyadh, Saudi Arabia

**Keywords:** mupirocin, cinnamon essential oil, nanostructured lipid carrier, topical delivery, antibacterial activity

## Abstract

**Backgrounds:**

The aim of the current study was to develop a distinctive nanolipid formulation, namely, nanostructured lipid carrier (NLC), which would deliver an antibacterial medication such as mupirocin (MP). Additionally, cinnamon essential oil (CEO), which is reported to exhibit antibacterial activity, was utilized in the development process in an attempt to improve the influence of MP.

**Methods:**

As a consequence, different MP–NLC formulations were developed using the central composite design (CCD) approach. One optimized formula was selected and incorporated within the pre-formulated gel matrix, providing the MP–NLC-gel formula for efficient topical application. MP–NLC-gel was assessed for its physical characteristics to check its suitability for topical application and evaluated for its *in vitro* drug release over 6 h. Furthermore, it studied the formulation for its stability at different conditions; 25°C ± 2°C and at 4°C ± 3°C for 6 months. Finally, the formulation was examined for its antibacterial performance against gram-positive and -negative bacteria.

**Results:**

The developed topical NLC-gel formulation demonstrated pH 5.8, viscosity 14,510 cP, and spreadability 58.1 mm, which were seemed to be satisfactory properties for successful topical application. The drug was released successfully for over 6 h with 52.9%. Additionally, it was stable in both storage conditions for 6 months since it displayed non-significant variations in its evaluated characteristics compared to those of fresh preparation. Ultimately, the developed gel formulation could inhibit the growth of different bacterial strains, especially gram-negative strains.

**Conclusion:**

To sum up, these findings would demonstrate the efficiency of NLC prepared with CEO and incorporating MP to be a promising antibacterial lipid nanocarrier.

## 1 Introduction

Nanotechnology is an innovative strategy that paved the way for scientific advancement in different aspects, especially in the medical application field (Anjum et al., 2021). This was accomplished via developing certain nanocarriers in a nanosized scale and large surface area, which consequently would enhance drug solubility and bioavailability (Din et al., 2017). A wide range of nanocarriers have been developed; nevertheless, lipid nanocarriers have presented a great promise in the topical delivery of drugs (Navarro-Partida et al., 2021). Lipid nanocarriers would suggest a sustained release manner that is essential for effectively dealing with different dermatological disorders (Gupta et al., 2012). These lipid nanocarriers comprise nanoemulsions, liposomes, solid lipid nanoparticles, and nanostructured lipid carriers (NLCs). Notably, NLCs gained a lot of attention, owing to their great advantages over other lipid-based nanocarriers (Shehata et al., 2024). It was reported that NLCs greatly impact delivering of medications topically, owing to their biocompatibility and stability. Rationally, lipid nanocarriers typically consisted of a lipid core; however, in NLCs, the lipid core consisted of both solid (fat) and liquid (oil) lipids. As a result, a drug would dissolve in the liquid lipid and then be encapsulated into the solid lipid, providing a higher drug loading capacity and greater entrapment efficiency (Viegas et al., 2023). Of late, NLCs could entrap different pharmaceutical agents for achieving various influences such as hypolipidemic (Elsewedy et al., 2022a), anticancer (Hajipour et al., 2018), anti-inflammatory (Guilherme et al., 2019), analgesic (Khurana et al., 2015), and antibacterial drugs (Elsewedy et al., 2023). Moreover, NLCs could be used to deliver the medication via various routes including inhalation (Almurshedi et al., 2021), rectal (Alotaibi et al., 2021), oral (Alavi et al., 2022), parenteral (Ilić et al., 2023), and topical routes (Bujubarah et al., 2023).

Topical drug delivery is a quick and efficient strategy for directing the medication to a specific targeted site, which would maximize the effectiveness of the drug (Benson et al., 2019). It is considered a route of choice in the case of being inconvenient with other routes (Elsewedy et al., 2024). Skin disorders including microbial infections are most likely to be treated with topical formulations (Williamson et al., 2017). Antibacterial drugs are among the frequently used drug category for treating several disorders related to bacterial infection (Bokhtia et al., 2022). One of the most efficient antibacterial agents, especially for skin infections, is mupirocin (MP) (Gangwar et al., 2021). MP is a secondary metabolite, used as a topical antibiotic, which acts by binding to specific bacterial enzymes and preventing them from making proteins (Connolly et al., 2019). It treats infections caused mainly by methicillin-resistant *Staphylococcus aureus* (MRSA) (David et al., 2018).

Several literature reports stated that MP is an ideal antibacterial agent since it showed poor antibacterial effect against the normal skin flora. In addition, it is effective in the management of various skin disorders like impetigo. It can be applied as a burn therapy, for wound healing, and diabetic wound healing (Gangwar et al., 2021; Twilley et al., 2022). However, it was reported that a widespread use of MP resulted in staphylococci resistance (Twilley et al., 2022). Presently, microbial resistance is one of the prevalent risks to the world’s public health that should be managed (Prestinaci et al., 2015). One possible way to manage microbial resistance is to improve the efficacy of the available antibiotics and decrease their frequency of administration (Ahmed et al., 2020). This improvement in drug activity could be achieved via a combination therapy, in which the drug substance is used in combination with other substances of same pharmacological activity. For that reason, an effort was made to incorporate MP into certain nanocarriers such as NLC using other natural compounds in order to improve the antibacterial influence of the drug. Accordingly, the significance of that combination therapy was the expected enhanced antibacterial influence. Additionally, it regarded as an alternative therapy for fighting bacterial resistance. Moreover, it investigates how natural and synthetic compounds could complement one another to improve the therapeutic outcomes and possibly lower the adverse effects. Consequently, the idea of combination therapy using the nanotechnology approach should be considered a valuable addition in antibacterial therapy.

Consistent with obtaining safe and efficient formulations, natural products, like essential oils, were extensively used (Miryusifova et al., 2024; Karadağ and Omarova, 2024). Cinnamon essential oil (CEO) is obtained from the bark of the *Cinnamomum* tree and considered one of the essential oils that is known for its safety and efficacy (Lucas-González et al., 2023). It showed great influence as an antifungal and antiviral agent against COVID-19 in addition to its antioxidant and anticancer activities (Wang et al., 2023; Sadeghi et al., 2019; El-Meidany et al., 2023). Moreover, it was reported that CEO exhibited effective antibacterial activity, owing to the presence of trans-cinnamaldehyde, which is thought to be the only significant component in CEO (El Atki et al., 2019).

Herein, the objective of the present study was to develop NLC formulations using CEO and incorporating MP. To the best of our knowledge, this is the first NLC formulation prepared using CEO and carrying MP. For obtaining a formulation with good traits and higher quality, central composite design (CCD) was employed as a way for the quality by design (QbD) approach. Several MP–NLC preparations were synthesized using the proposed concentrations of the independent variables. Based on the obtained dependent variables and their adjustment toward certain desired constrains, the optimized MP–NLC formula was selected, characterized, and incorporated into gel preparation to be easily applied topically and explored for its antibacterial behavior.

## 2 Methods

### 2.1 Materials

MP was obtained as a gift sample from AVALON PHARMA (Middle East Pharmaceutical Industries Co. Ltd., Riyadh, Saudi Arabia). CEO was bought from NOW^®^ Essential Oils (NOW Foods, Bloomingdale, IL, United States). Polysorbate 80 (Tween 80), hydroxypropyl methylcellulose (HPMC), and stearic acid were procured from Sigma-Aldrich Co. (St Louis, MO, United States). Diethylene glycol monoethyl ether (Transcutol^®^ P) was purchased from Gattefosse SAS (Saint-Priest Cedex- France). All other solvents and chemicals were of analytical grade.

### 2.2 Central composite experimental design

For obtaining a product with the finest properties and of highest quality, CCD was utilized, which is the most commonly used factorial design of response surface methodology (Bhattacharya, 2021). The study was based on specifying definite variables to be the independent factors that exerted their influence on certain dependent variables known as the response (Elsewedy et al., 2022b). Two independent factors were assigned, lipid phase and surfactant concentration, represented by X1 and X2, respectively. On the other side, the studied dependent responses were particle size R1 and percentage of encapsulation efficiency (EE) R2. The study was adopted using Design-Expert version 12.0 software (Stat-Ease, Minneapolis, MN, United States), producing 10 formulations, as displayed in Table 1. Through CCD, statistical analysis of all obtained data was performed using analysis of variance (ANOVA) to validate the adequacy of the model. Additionally, some generated graphs were created to help in confirming the obtained results. Furthermore, the design produces polynomial mathematical equations that could prove the relation between the independent variables and the studied dependent response as follows:

R=bo+b1 X1+b2 X2+b12 X1 X2+b11 X12+b22 X22,

where R characterizes the dependent variable, whereas b0 represents the intercept; b1, b2, b12, b11, and b22 are the regression coefficients. X1 and X2 signify the main factors; X1 and X2 indicate the interactions between the main factors, and X12 and X22 point toward the polynomial terms.

**TABLE 1:** Experimental runs developed by CCD showing different independent factors and their observed responses.

Run	Independent variables		Dependent responses		PDI
	X1: lipid phase %	X2: surfactant %	R1: P. size nm	R2: EE %	
NLC 1	20	5	299 ± 4.0	89 ± 3.1	0.36 ± 0.04
NLC 2	10	7.5	146 ± 2.6	58 ± 3.4	0.23 ± 0.026
NLC 3	15	5	234 ± 2.1	77 ± 2.7	0.36 ± 0.025
NLC 4	15	5	238 ± 3.6	79 ± 3.5	0.30 ± 0.026
NLC 5	20	7.5	281 ± 3.2	87 ± 2.8	0.29 ± 0.025
NLC 6	20	2.5	317 ± 4.6	93 ± 4.2	0.39 ± 0.021
NLC 7	15	2.5	256 ± 3.5	83 ± 3.2	0.29 ± 0.025
NLC 8	10	2.5	184 ± 2.6	64 ± 1.9	0.22 ± 0.030
NLC 9	15	7.5	227 ± 3.2	74 ± 2.9	0.29 ± 0.021
NLC 10	10	5	162 ± 2.6	61 ± 1.9	0.23 ± 0.025

### 2.3 Development of MP–NLC

Ten NLC formulations incorporating MP were prepared using the percentages proposed by CCD software, as displayed in Table 1. The fabrication process was carried out using the melt emulsification–ultrasonication method that was stated previously (Elsewedy et al., 2022a). Based on the performed preliminary studies to select the best ratio between the solid lipid and liquid lipid phase, it was revealed that 2:8 was the optimum. Initially, the specified amount of stearic acid (the solid lipid) was melted at a temperature higher than its stated melting point (69.3°C). In addition, the quantified amount of CEO representing the liquid lipid was heated at the same temperature and mixed with the melted stearic acid to form the lipid phase. A measure of 1 mL of Transcutol^®^ P acting as a solubilizing agent and penetration enhancer and the required amount of MP were added to the lipid phase and mixed well until a homogenous mixture was formed. On the other hand, the aqueous phase up to 10 mL was prepared by adding the determined amount of Tween 80 (surfactant) and heated to the same temperature. The aqueous phase was gradually added to the melted lipid phase with constant stirring until pre-emulsion was obtained. Using the Ultra-Turrax homogenizer (IKA-T25; Germany), the pre-emulsion was homogenized at 10.000 rpm for 5 min, followed by sonication at 10.000 rpm using the probe sonicator XL-200, Qsonica (New town, CT; United States), until reaching the proper particle size.

### 2.4 Evaluation of MP–NLC characteristics

#### 2.4.1 Particle size and polydispersity index (PDI) analysis

The dynamic light scattering approach was used to determine the particle size of the MP–NLCs that were formulated. Using a disposable cuvette, approximately 5 µL of each formulation was diluted with 3 mL of distilled water to assess the particle size and size distribution (PDI). A Malvern zetasizer (Nanoseries, zs; Malvern Instruments, Malvern, United Kingdom) was used for this investigation at 25°C (Shehata and Elsewedy, 2022).

#### 2.4.2 Drug encapsulation efficiency (EE %)

As stated previously (Elsewedy et al., 2022b), centrifugation was used to measure the percentage of MP encapsulated within the NLC. In short, a sample of the NLC formulation under examination was put into a centrifuge tube, namely, Amicon^®^ Ultra-4 (Ultracel-10K), and the tube was centrifuged at 4°C and spun at 3,000 rpm for 1 hour. From the filtrate, free MP was collected, diluted, and measured using a spectrophotometer (UV Spectrophotometer, JENWAY 6305) at λ_max_ 222 nm. The following equation was applied to calculate the EE:

$$\%\text{ EE}=\left(\left(\text{Total amount of MP}-\text{Free amount of MP}\right)/\text{Total amount of MP}\right)\times 100.$$

### 2.5 Fourier transform infrared analysis

Checking the possibility of interaction between the drug and NLC excipients was very essential. An FTIR spectrophotometer (FTIR spectrophotometer, SHIMADZU, IRAFFINITY-1S, Japan) was utilized for such investigation using the KBr disc method. The KBr plate was prepared and compressed, and different samples were added over the disc and left to dry in vacuum. The spectra were recorded within a scale between 4,000 and 400 cm −1 for free MP, blank NLC, and optimized MP–NLC (Elsewedy et al., 2022a).

### 2.6 Development of the topical MP–NLC gel

In order to apply the topical formulation easily and conveniently, viscous formulations are used most preferably. In view of that, the optimized MP–NLC was combined with 10 g of a pre-formulated gel base to provide 2% MP–NLC gel. The gel base formulation was developed by simply scattering the gelling agent (4% w/w HPMC) over distilled water and kept stirring with a magnetic stirrer (Jeio Tech TM-14SB, Medline Scientific, Oxfordshire, United Kingdom) until the HPMC-gel base was produced (Kakkar et al., 2018). The same method was utilized to formulate the NLC-gel without MP, termed as the blank NLC-gel, which was required for further investigation.

### 2.7 Surface morphology

The morphology of the fabricated MP–NLC-gel was examined via a microscopic technique by scanning electron microscopy (SEM) (Hitachi-S-3700N, Japan). A sample of the formulation was diluted and put on metal stubs then coated with gold and scanned. The sample was analyzed at 10 kv using 5K magnification (Elsewedy et al., 2022a).

### 2.8 Visual examination and pH determination of the MP–NLC-gel

Different parameters were examined in the interest of evaluating the fabricated NLC-gel, including visual examination and pH determination. Visual inspection of the formulation was performed for its appearance, homogeneity, and consistency. Moreover, the pH value is very essential for proving the safety of the formulation upon topical application over the skin. Thus, a calibrated pH meter (MW802, Milwaukee Instruments, Szeged, Hungary) was utilized for such measurement (Elsewedy et al., 2022b).

### 2.9 Viscosity profile of the MP–NLC-gel

Since viscosity is very essential for proper application of a topical formulation, the MP–NLC-gel was examined for its viscosity using a Brookfield viscometer (DV-II + Pro, Middleboro, United States). The estimation was performed using spindle 63 at a temperature of 25°C and rotation 0.5 rpm (Shehata and Elsewedy, 2022).

### 2.10 Spreadability of the MP–NLC-gel

Spreadability is another important factor that shows how evenly the formulation could spread over the skin. To test it, a sample of approximately 0.5 g of the gel base was added between two glass plates having the diameter 25 cm × 25 cm. Then, a load weighing 500 g was placed over the upper plate for 2 min. Spreadability would be determined by measuring the diameter of the spreading area (Dantas et al., 2016).

### 2.11 Drug release study

Franz diffusion cells (Logan Instruments Corp., FDC-6, Somerset, NJ, United States) were utilized for the *in vitro* release study of MP formulations. In brief, freshly prepared phosphate buffer pH 5.5 was put into the receptor chamber of the diffusion cell. Both the donor and receptor chambers were separated from each other by a cellulose acetate cellophane membrane (MWCO 2,000–15,000). The samples were removed at the scheduled times (0.25, 0.5, 1, 2, 3, 4, 5, and 6 h) and replaced with equal volumes of new buffer solution at the appropriate intervals to keep the volume constant during testing. Following the appropriate dilutions, the samples were subjected to a UV-visible spectrophotometer (JENWAY 6305, Bibby Scientific Ltd., Staffs, United Kingdom) set to ƛ_max_ 222 nm to determine the drug content (Bujubarah et al., 2023). Each experiment was performed in triplicate with mean value ± SD.

### 2.12 Kinetic study

For investigating the mechanism by which the drug was released from the optimized MP–NLC-gel, different kinetic models were followed to conclude the best-fit model. The studied models were zero-order kinetic that reflects the percentage of drug released against time; first-order kinetic that reveals the log percentage of the drug remaining against time; Higuchi’s model, which shows the percentage of drug released against the square root of time; and Korsmeyer–Peppas kinetic model that demonstrates the log percentage of the drug released against log time (Shehata et al., 2022).

### 2.13 Long-term stability study

The study was carried out for the formulated MP–NLC-gel over a period of 6 months of storage in accordance with International Conference on Harmonization (ICH) guidelines. The sample was kept in stability chambers in a closed bottle at room temperature (25°C ± 2°C) and at refrigeration (4°C ± 3°C). The sample was analyzed for its pH, viscosity, and any indication of separation. All parameters were evaluated in triplicate each month, and the results were reported as mean ± SD (Elsewedy et al., 2022c).

### 2.14 Antibacterial study

The antibacterial performance of the examined formulations was assessed via a disk diffusion method to assess the zone of inhibition. The study was performed using different bacterial strains provided from the American Type Culture Collection (ATCC). A strain of *Staphylococcus aureus* (ATCC 29213) was used as a representative gram-positive bacteria, and *Escherichia coli* (ATCC 25922) was used as demonstrative gram-negative bacteria. In a petri dish, Mueller–Hinton agar was prepared and dispensed to be a culture media for the bacteria; then, three wells of 6-mm diameter were made in each Petri dish. MP–NLC-gel, blank NLC-gel, and the marketed product (Bactroban^®^) were the investigated formulations that were poured into the wells. Each Petri dish was incubated for 24 h at 37°C ± 1°C, and then, the inhibition zone diameter was measured to provide an indication for the antibacterial activity of the formulation. Each experiment was performed in triplicate with mean value ± SD.

### 2.15 Statistical data analysis

Data were expressed as mean ± standard deviation (SD). The significant level was detected if *P* < 0.05. Student’s t-test was applied to identify the statistical differences between the groups. All statistical analyses were confirmed by SPSS statistics software, version 9 (IBM Corporation, Armonk, NY). One-way analysis of variance (ANOVA) was executed using Design-Expert version 12.0 software (Stat-Ease, Minneapolis, MN, United States).

## 3 Results

### 3.1 Optimization study

Applying the QbD approach in the optimization process represented by CCD has become a prevalent strategy for obtaining an optimized product with favorite characteristics (Kumbhar et al., 2020). CCD software provided a matrix of 10 formulations (NLC1–NLC10) using specific independent factors that showed their influence on dependent responses, as summarized in Table 2. ANOVA was helpful in analyzing all data responses, which is very essential for fitting the design model.

**TABLE 2:** ANOVA statistical data generated from CCD software for particle size response.

Source	R_1_	
	F-value	*p*-value
Model	564.31	<0.0001*
X_1_	2,636.45	<0.0001*
X_2_	170.52	0.0002*
X_1_ X_2_	0.0964	0.7717
X_1_^2^	13.64	0.0210*
X_2_^2^	2.32	0.2020
Lack of fit	1.39	0.5406
R^2^ analysis		
R^2^	0.9986	
Adjusted R^2^	0.9968	
Predicted R^2^	0.9880	
Adequate precision	67.8887	
Model		
Remark	Quadratic	

X_1_, lipid phase (%); X_2_, surfactant; R_1_, Particle size (nm); *, significant *P* < 0.05.

### 3.2 Analysis of dependent response

#### 3.2.1 Influence of the independent factors on the particle size response

As apparent in Table 1, particle size was measured for all MP–NLC preparations and showed values between 146 ± 2.6 and 317 ± 4.6 nm with a PDI value less than 0.5. The result demonstrated that all formulations exhibited a narrow range of sizes, which suggest the stability of the formulation (Danaei et al., 2018). It was very obvious that both independent factors, X_1_ and X_2_, exerted a marked influence on the investigated particle size response (R_1_). A direct positive relationship was detected between the lipid phase concentration (X_1_) and the particle size (R_1_), where increasing (X_1_) showed a consistent increase in (R_1_). This could be attributed to particles’ coalescence and aggregation that might happen upon using a higher lipid concentration, leading to bigger particles, besides a possible increase in the dispersed phase (Elsewedy et al., 2022a). On the contrary, a higher Tween 80 concentration (X_2_) would decrease the particle size while using the same concentration of the lipid phase. The rationale behind that is the action of the surfactant in lowering the interfacial tension between the lipid phase and aqueous phase (Sarheed et al., 2020). Furthermore, the surfactant could arrange as a layer around the particles, which prevents them from being aggregated together, and thus keep the particles small (Miyazawa et al., 2021). The following mathematical equation confirmed the obtained results since there was a positive sign in front of factor X_1_ that confirmed its direct effect on the response. However, the negative sign in front of X_2_ indicated its antagonistic influence on the examined response R_1._

$$R_{1}=237.143+67.5\;X_{1}-17.1667\;X_{2}+0.5\;X_{1}\;X_{2}-7.78571\;X_{1}^{2}+3.21429\;X_{2}^{2}.$$

Referring to the statistical analysis report displayed in Table 2, the model F-value was verified to be significant as it was 564.31, which has a *p*-value less than 0.0001. Moreover, most of model terms (X_1_, X_2_, and X_1_^2^) significantly affected the particle size as long as their *P*-values were less than 0.05. Another important indicator for model fitting is the lack of fit, which is recommended to be non-significant. As shown in Table 2, the value of lack of fit was 1.39 with corresponding *p*-values of 0.5406, which denotes non-significant lack of fit. Furthermore, a linear correlation was observed between the predicted and the actual values of X_1_ response since the values of the predicted R^2^ and adjusted R^2^ were very close to each other, indicating a reasonable agreement with each other. The value of adequate precision was 67.8887, which is an adequate signal that navigates the design space.

**FIGURE 1:**
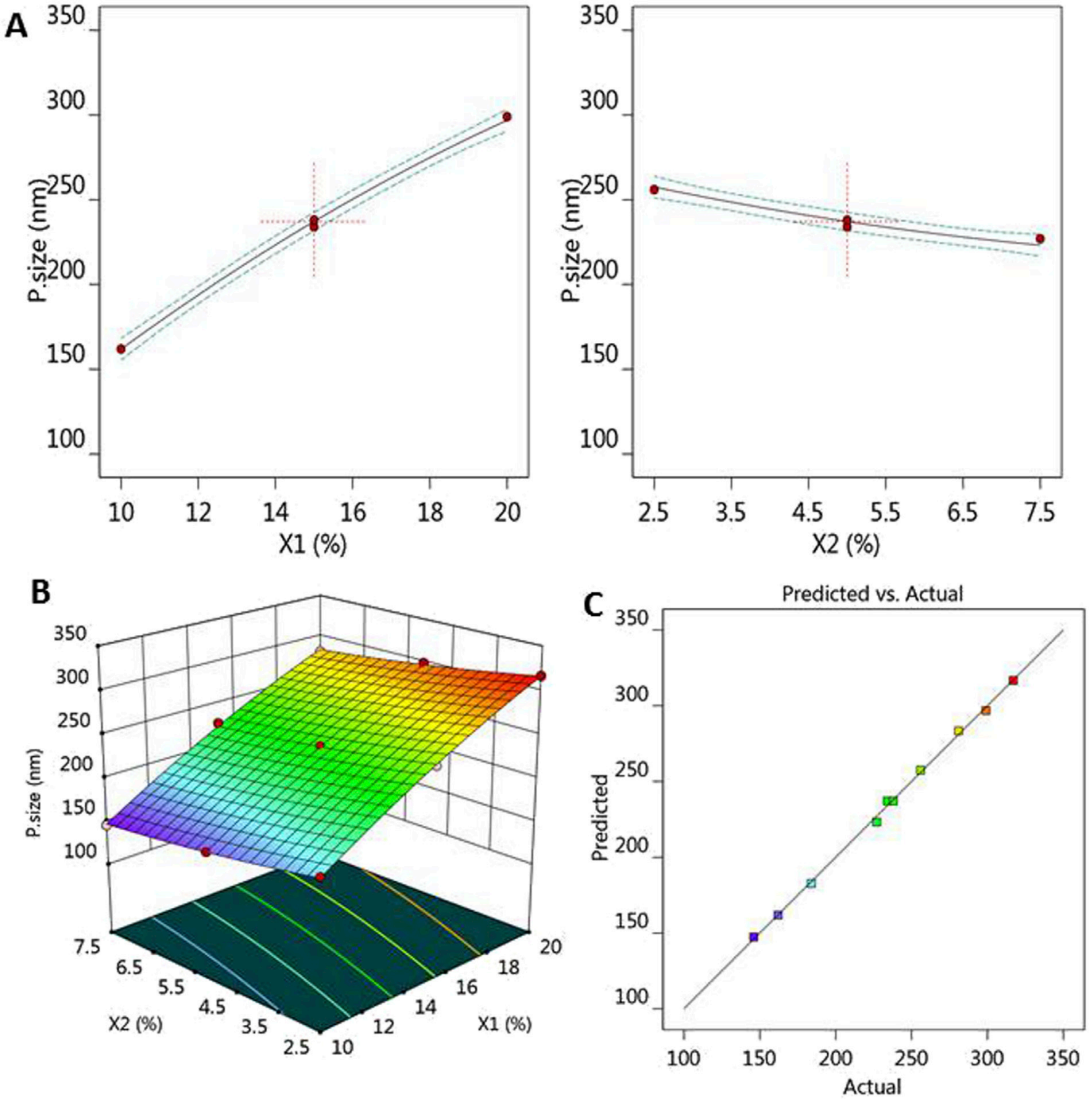
**(A)** All-factor graph showing the influence of independent variables X_1_ and X_2_ on the investigated particle size R_1_. **(B)** 3D-surface plot displaying the influence of independent variables X_1_ and X_2_ on the studied particle size R_1_. **(C)** Linear correlation plot between the predicted and actual values presenting the effect of independent variables X_1_ and X_2_ on the investigated particle size R_1_.

The previous results were further illustrated through several graphs produced by the software application that emphasized the effect of the selected independent variables on the observed response R1. As shown in Figure 1A, all factor graphs for each independent variable were plotted against the particle size response. The graph established that the particle size of the fabricated MP–NLCs increased upon the increment of independent variable X_1_ and upon decreasing X_2_. Additionally, the significant effect of the lipid phase concentration (X_1_) and surfactant concentration (X_2_) on the particle size (R_1_) of MP–NLCs is clearly illustrated in Figure 1B related to the 3D-response surface plot. Furthermore, the linear correlation between the predicted and the actual values of the response is verified in Figure 1C.

#### 3.2.2 Influence of the independent factors on EE response

Entrapment of drug inside the formulation is another parameter essential to be evaluated. Accordingly, EE of the fabricated MP–NLC formulations was estimated and seemed to be ranged between 58% ± 2.4% and 93% ± 4.2%. Similar to particle size, the studied independent variables strongly influence the EE response (R_2_). According to the obtained data, it was apparent that increment of the lipid phase concentration (X_1_) would positively influence the EE, resulting in a corresponding increase in R_2_. This finding was supposed to be due to the MP lipophilic property as it preferred to be dissolved in the melted lipophilic phase, thus enhancing its entrapment (Anwar et al., 2020). On the other hand, the influence of the independent variable (X_2_) on the EE response R_2_ was indirect; hence, increasing the surfactant concentration would decrease the percentage of the entrapped drug. This was due to the small particle size obtained by increasing the surfactant concentration as a small particle size could not accommodate for entrapping a higher amount of the drug (Shehata and Elsewedy, 2022). Further demonstration for data was clarified by the mathematical equation. It was noted that X_1_ possessed a positive sign, indicating a positive synergistic effect. However, X_2_ carried a negative sign that signified an opposite influence.

$$R_{2}=78+14.3333\;X_{1}-3.5\;X_{2}+2.88312e-14\;X_{1}\;X_{2}-3\;X_{1}^{2}+0.5\;X_{2}^{2}.$$

From the CCD report, the model F-value was proven to be significant since it showed the value of 199.08, which has a *p*-value less than 0.0001, as clearly shown in Table 3. In addition, it was noted that X_1_, X_2_, and X_1_^2^ were significant model terms with *p*-values less than 0.05, confirming their significant effect on EE. Regarding the lack of fit value, it showed an F-value 0.5556 with the *p*-value 0.7278, indicating a non-significant value that is highly recommended. Likewise, a reasonable agreement was detected between the predicted and adjusted R^2^ since the difference between them is less than 0.2. Therefore, a linear correlation was observed between the predicted and the actual values of X_2_ response. The adequate precision of X_2_ response was 39.8765, which is an adequate signal that navigates the design space.

**TABLE 3:** ANOVA statistical data generated from CCD software and fit statistics of R^2^ analysis for EE response.

Source	R_2_	
	F-value	*p*-value
Model	199.08	<0.0001
X_1_	924.50	<0.0001
X_2_	55.13	0.0018
X_1_ X_2_	0.0000	1.0000
X_1_^2^	15.75	0.0166
X_2_^2^	0.4375	0.5445
Lack of fit	0.5556	0.7278
R^2^ analysis		
R^2^	0.9960	
Adjusted R^2^	0.9910	
Predicted R^2^	0.9687	
Adequate precision	39.8765	
Model		
Remark	Quadratic	

X_1_, lipid phase (%); X_2_, surfactant; R^2^, EE (%); *, significant *P* < 0.05.

**FIGURE 2:**
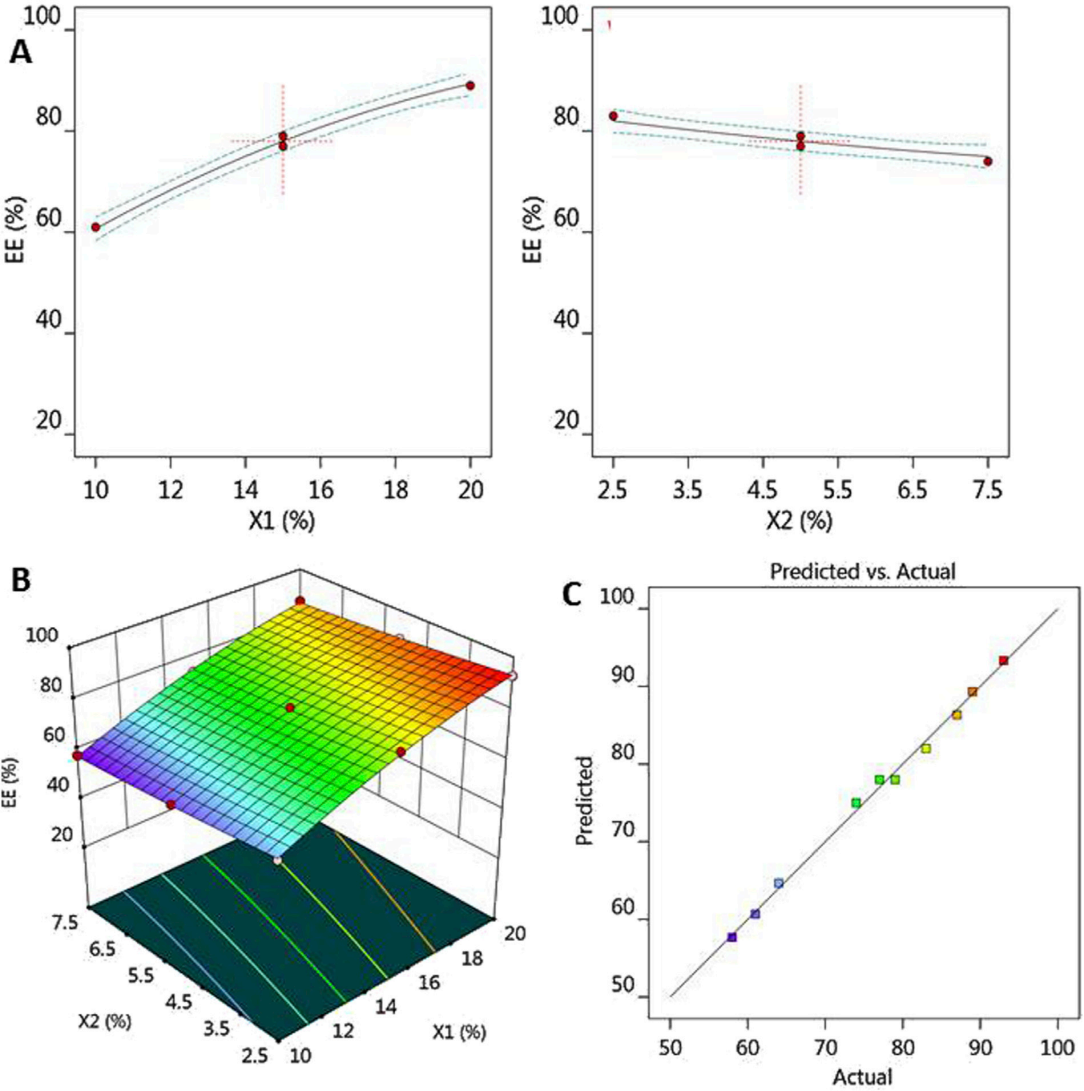
**(A)** All-factor graph showing the influence of independent variables X_1_ and X_2_ on the investigated EE R_2_. **(B)** 3D-surface plot screening the effect of independent variables X_1_ and X_2_ on the examined EE R_2_. **(C)** Linear correlation plot between the predicted and actual values presenting the influence of independent variables X_1_ and X_2_ on the investigated EE R_2_.

It is depicted in Figure 2A that all factor graphs that explained the positive influence of the lipid phase concentration (X_1_) on R_2_ response since increasing (X_1_) would result in the subsequent increase in EE. On the contrary, the surfactant concentration (X_2_) exerted an inverse correlation with R_2_ response where their increase resulted in decreasing EE. Additionally, Figure 2B shows the 3D-surface plot confirming the influence of both independent variables on the investigated response R_2_. Moreover, Figure 2C confirmed the linear correlation between the predicted and actual values allied to EE. In the same manner, a reasonable agreement between the predicted and adjusted R^2^ value was observed as the difference between them was less than 0.2. This observation emphasized the linear correlation between the predicted and actual values.

### 3.3 Optimization and validation of data

**FIGURE 3:**
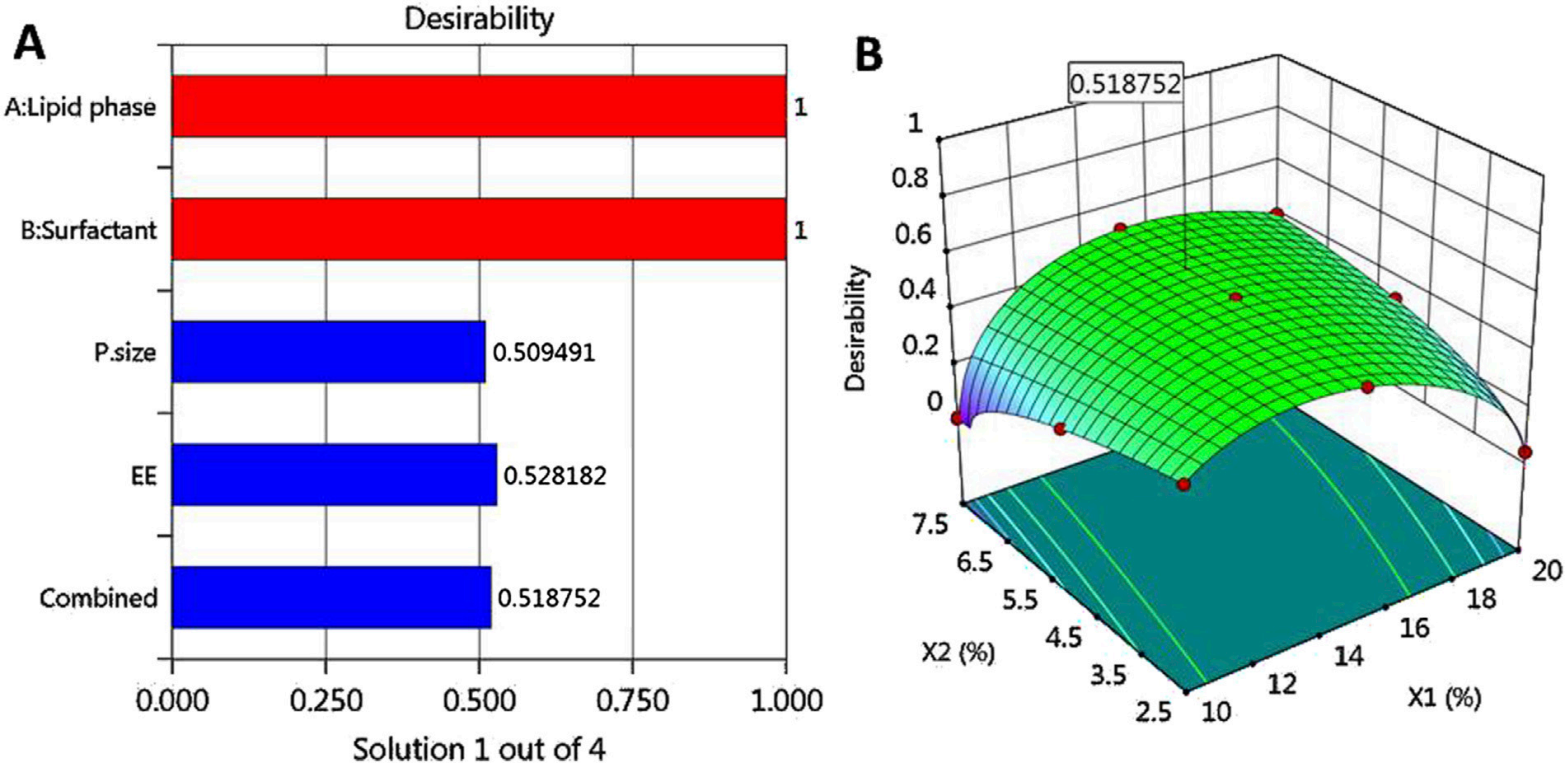
Optimization showing the desirability value via **(A)** bar graph and **(B)** 3D surface plot.

**FIGURE 4:**
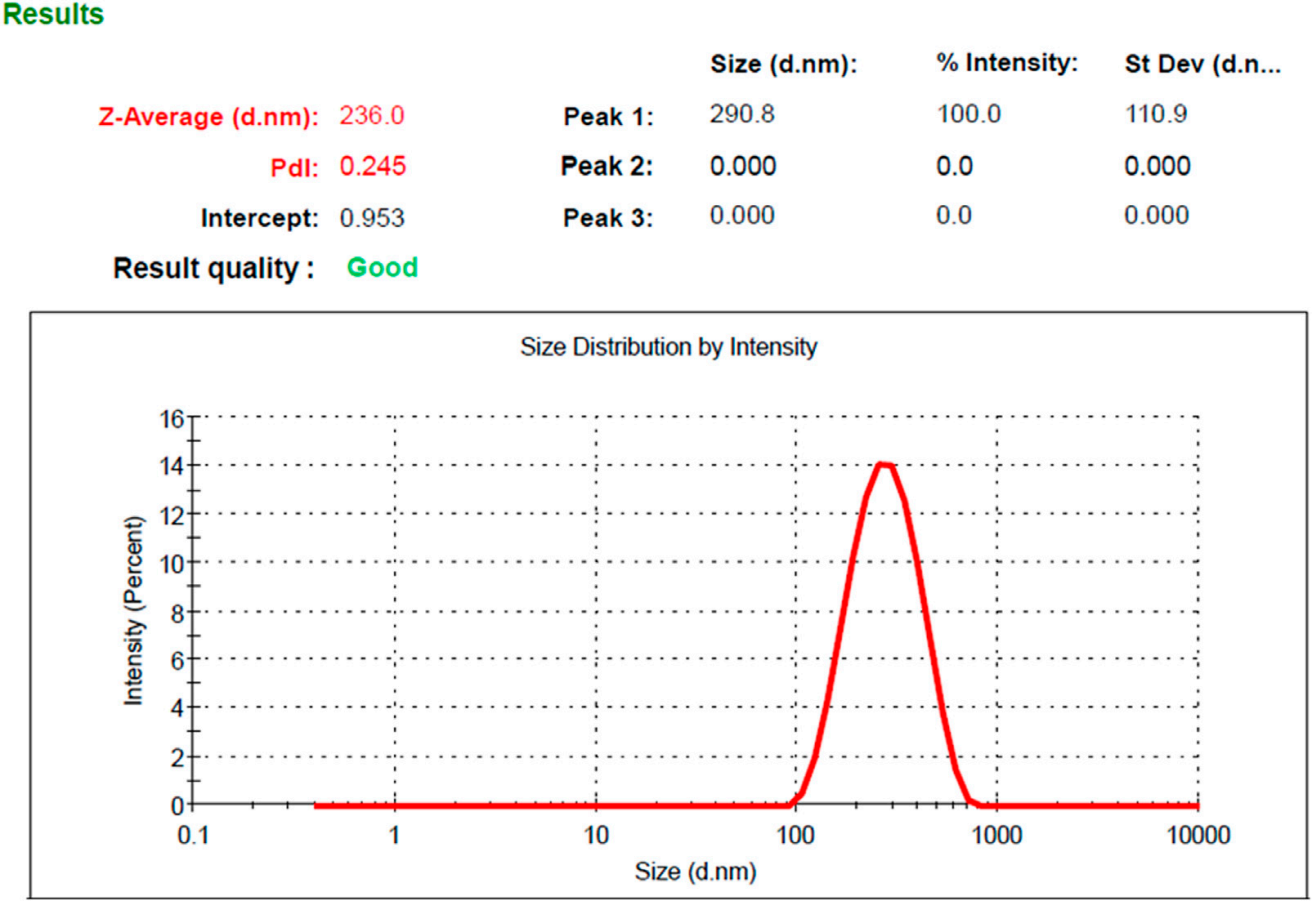
Particle size of the optimized MP–NLC formulation.

Based on the optimization process and the collected data from numerical optimization criterion, the optimum MP–NLC formula was elected. The independent factors were kept in range, while the dependent responses were directed toward minimizing X_1_ and maximizing X_2_ response. The report of the numerical optimization offered different solutions with different desirability values. The highest desirability value was selected (0.519), predicting the suggested concentration of X_1_ and X_2_, as displayed in Table 4 and Figure 3. Subsequently, a new MP–NLC formulation was fabricated, and the optimized formula was considered. It was noted that the predicted and the observed values of response were very similar. As shown in Figure 4, the particle size of the optimized MB–NLC preparation was measured.

**TABLE 4:** Predicted versus observed value for the optimized MP-NLC formulation.

Dependent response	Predicted value	Observed value
Particle size (nm)	229.87 ± 3.22	236.0 ± 3.6
EE (%)	76.48 ± 1.15	78.20 ± 2.70

### 3.4 FTIR spectroscopy

**FIGURE 5:**
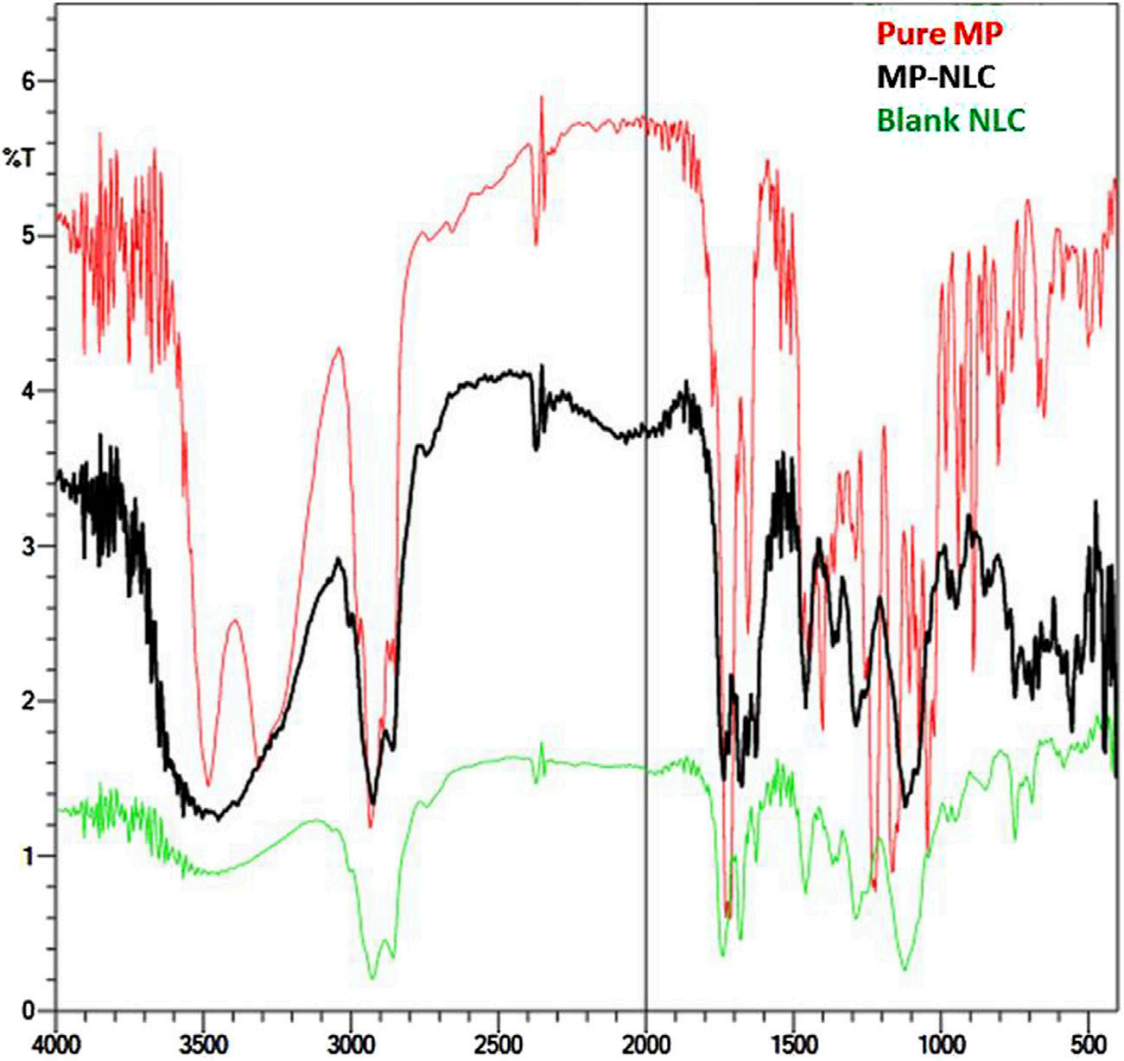
FTIR spectra analysis of pure MP, free NLC, and optimized MP–NLC formulation.

As depicted in Figure 5, infrared spectroscopy was used to investigate potential chemical interactions between the drug and other ingredients in the formulation. The spectra showed distinguishing peaks for pure MP, blank NLC, and MP–NLC. The following peaks were identified at 3,500–3,300 cm^−1^ (O–H), 2,920–2,860 cm^−1^ (CH_2_CH_2_− stretching), 1,730 cm^−1^, 1,710 cm^−1^ (C=O), 1,250 cm^−1^, and 1,120 cm^−1^ (C–O) in the observed spectra of pure MP. The FTIR spectra of blank NLC and MP–NLC revealed a strong broadband at 2,900 cm^−1^, which can be attributed to O–H stretching, resulting from intermolecular hydrogen. The findings imply that there are no interactions between the drug and the excipients, proving that the excipients are appropriate to be included into NLC. The results were in line with the findings of Shinde et al, who suggest that no interaction was detected between mupirocin and the content of the developed NLC (Shinde et al., 2022).

### 3.5 Surface morphology

**FIGURE 6:**
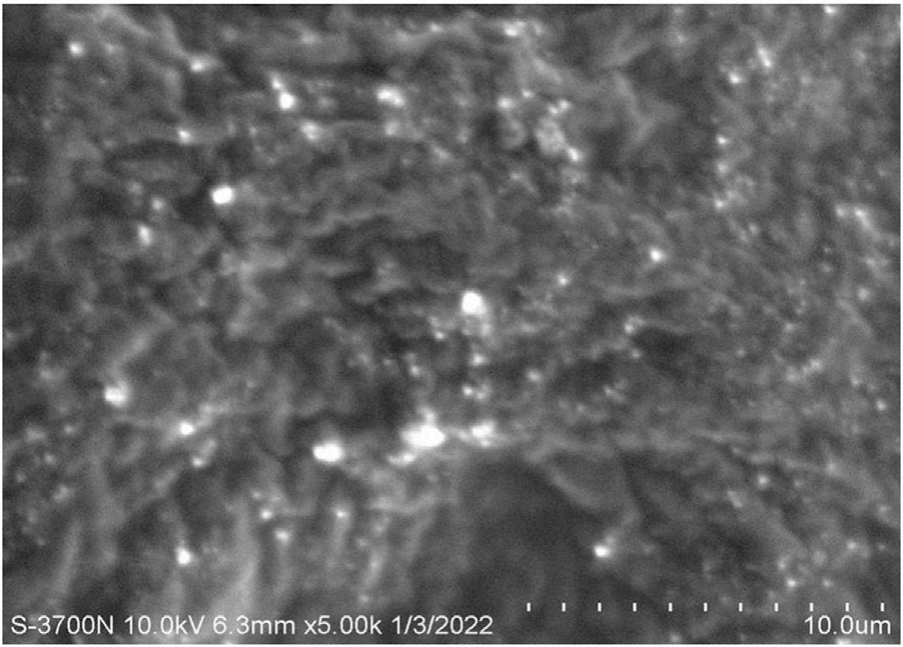
Scanning electron microscope image demonstrating the morphology of the MP–NLC-gel.

Morphological evaluation of the fabricated MP–NLC-gel formulation was carried out utilizing SEM. As shown in Figure 6, it was noticeable that there were small spherical vesicles related to the nanolipid formula dispersed into a porous network of the formed hydrogel.

### 3.6 Visual examination and pH determination of the MP–NLC-gel

Based on the optimizing process, the optimum NLC preparation containing MP was fabricated and incorporated into a pre-prepared HPMC gel-base. Consequently, MP–NLC-gel was obtained, and upon visual examination, it seemed to be homogenous, uniform, smooth, and stable without any clue for phase separation. The MP–NLC-gel was examined for its pH value, which is a substantial parameter that should be evaluated for topical preparations to indicate whether the formula is irritant or not. The pH of the MP–NLC-gel was found to be 5.8 ± 0.46, which is close to the normal pH of the skin. This finding ensures the safety of the formulation, following topical application. The result was in agreement with Lukić *et al*., who stated that the pH of the topical formulations was found to be within the range of 4–6 (Lukić et al., 2021).

### 3.7 Viscosity profile of the MP–NLC-gel

Referring to the formulation viscosity, it is a requisite requirement to be measured since it influences the drug diffusion from the formulation and, consequently, reflects the *in vitro* release behavior (Hassan and Hasary, 2023). The viscosity of the MP–NLC-gel was 14,510 ± 1,026 cP, which is presumed to be in an acceptable range for topical application (Shehata et al., 2022). This was in accordance with another study that proved the viscosity of a topical curcumin and caffeine mixture gel to be 12,329 Cp (Iriventi et al., 2020).

### 3.8 Spreadability of the MP–NLC-gel

Regarding formulation spreadability, it is very important to identify how simply and conveniently the topical preparation would spread over the affected area. Once the topical preparation spreads easily and consistently, the patient convenience can be achieved. Accordingly, spreadability of the examined formulation was estimated to be 58.1 ± 2.4 mm, which was established to be distinctive. The obtained result of spreadability was very similar to that of Bayoumi et al. (2022), where spreadability of the HPMC topical gel of ectoine was approximately 65 mm.

### 3.9 Drug release study

**FIGURE 7:**
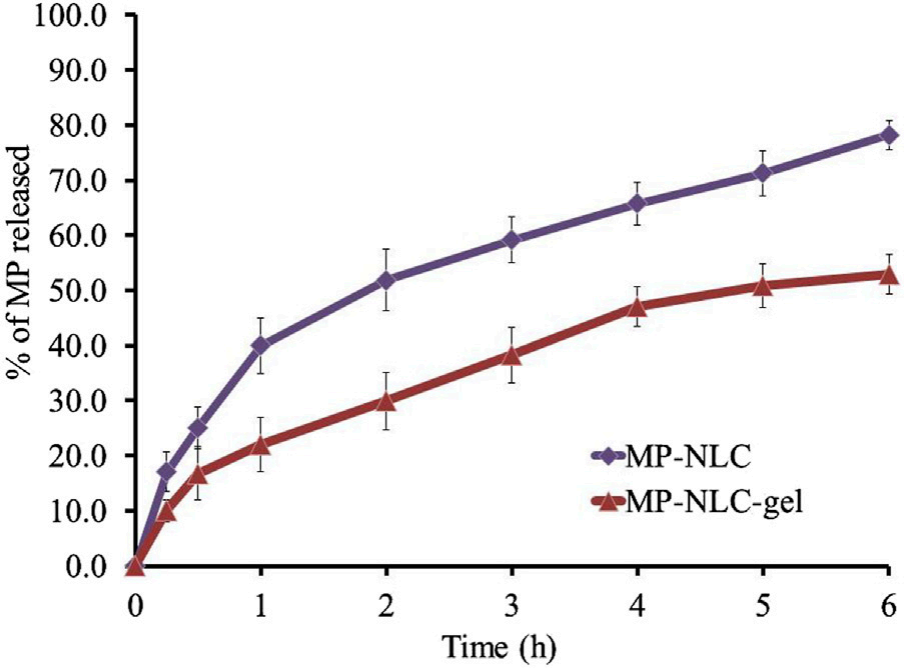
*In vitro* drug release profile of MP from the MP–NLC-gel formulation compared to optimized MP–NLC.

The MP release pattern from the optimized NLC and NLC-gel was adopted and compared, as depicted in Figure 7. It was obvious that following 180 min, the amount of MP released from the optimized MP–NLC was 78.20% ± 2.70%. On the other side, following 180 min, the release from the NLC-gel formulation reached 52.9% ± 3.6%. This is presumed to be due to the enclosure of MP within the nanolipid formula and further into the gel base. This definitely would result in taking a longer time for the drug to be released from the lipid core out of the carrier due to passing through many layers (Khan et al., 2023). Moreover, the high viscosity of the NLC-gel formulation would slow the rate of drug release (Binder et al., 2019).

### 3.10 Kinetic study

In view of the kinetic release study, a curve was plotted between the amount of drug released versus time, and the utmost value of (R^2^) along with the most linear plot was determined. The study exhibited that MP release obeyed the Higuchi kinetic mechanism since such a model achieved the highest value of R^2^ (0.9874 and 0.9948) for NLC and NLC-gel formulations, respectively, as displayed in Table 5. Additionally, when the drug was released from a matrix-type formulation like the nanolipid matrix, it is said to follow Higuchi kinetic modeling. Moreover, it is remarkable that the drug dissolution from transdermal systems always follows Higuchi kinetic modeling (Damodharan, 2020).

**TABLE 5:** Drug release kinetics from studied formulations.

Formulation	R^2^ value			
	Zero-order kinetic	First-order kinetic	Higuchi kinetic	Korsmeyer–Peppas kinetic
Optimized MP–NLC	0.8755	0.7889	0.9874	0.9848
MP–NLC-gel	0.927	0.8463	0.9948	0.9923

### 3.11 Long-term stability study

**FIGURE 8:**
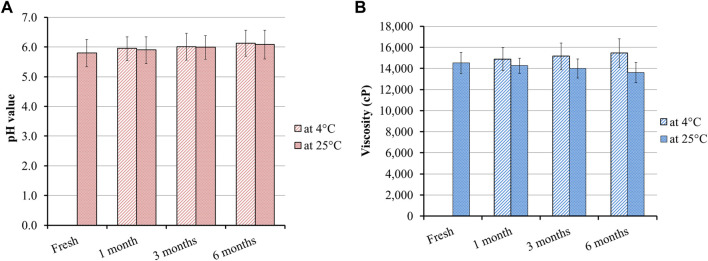
Stability of the MP–NLC-gel, following 1, 3, and 6 months storage at 25°C ± 2°C and at 4°C ± 3°C in terms of **(A)** pH and **(B)** viscosity compared to the fresh preparation.

Studying the stability of the developed MP–NLC-gel formulation was performed in terms of its physical appearance in addition to different parameters such as pH and viscosity. The study was conducted to investigate the influence of the storage period for 1, 3, and 6 months storage and the storage temperature at two different environments, 25°C ± 2°C and at 4°C ± 3°C, as illustrated in Figure 8. It was obvious that there were no significant changes detected in the assessed parameters, following 1, 3, and 6 months storage at the two predetermined conditions when compared to the fresh formulation. These findings highlighted the formerly identified fact regarding NLC stability as a result of its structure. Meanwhile, NLC has a lipid core sheltered with a surfactant that offers better physical stability to the formulation during its storage (Huang et al., 2017).

### 3.12 Antibacterial study

**FIGURE 9:**
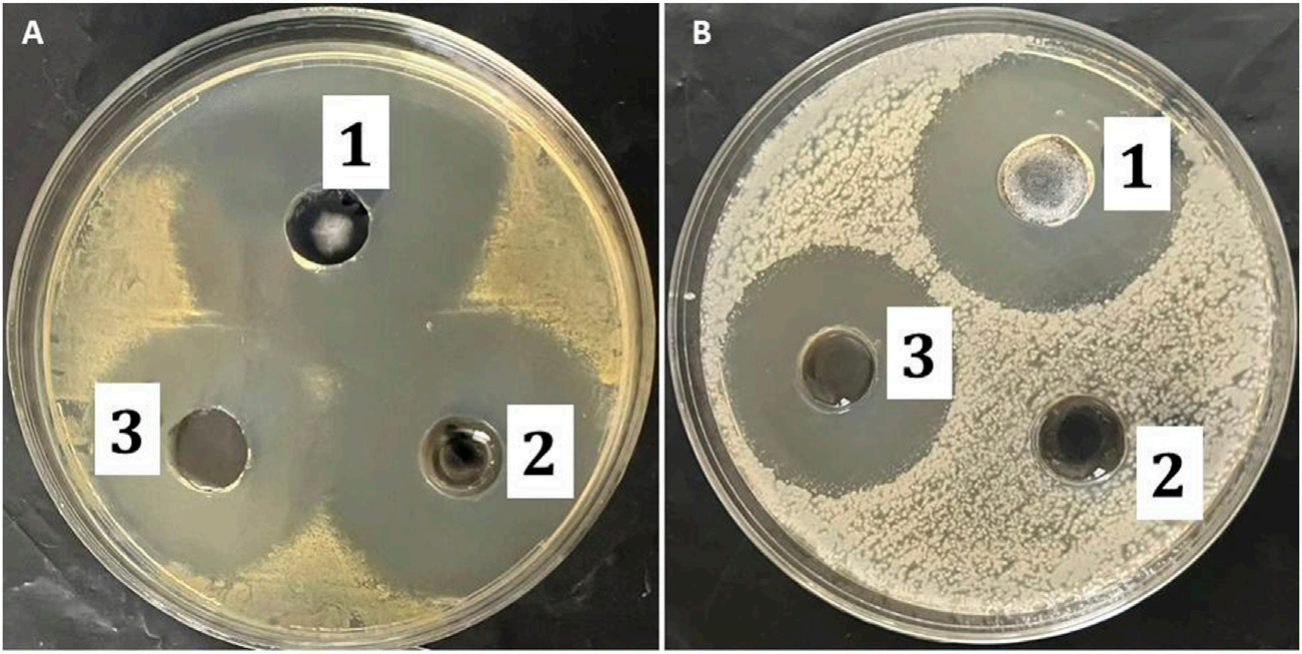
Inhibition zone diameter produced by the studied formulations: 1) MP–NLC-gel, 2) marketed product (Bactroban^®^), and 3) blank NLC on different bacteria; **(A)** *Staphylococcus aureus* and **(B)** *E. coli*.

The antibacterial activity of the MP–NLC-gel against several bacterial strains was analyzed, and the results are shown in Table 6; Figure 9. The study was accomplished by measuring the inhibitory zone that might be formed as a result of the formulation’s action on bacteria. The inhibitory zone was measured for the MP–NLC-gel; blank NLC-gel, and commercial product (Bactroban^®^). Compared to the blank NLC-gel, the MP–NLC-gel demonstrated a considerable inhibitory zone, indicating its high level of activity against *Staphylococcus* bacterial strains, *P* < 0.05. Remarkably, a notable reduction of bacterial growth in the media was observed by blank NLC-gel, which is most likely because CEO was included in the formulation. This result would highlight previously published information about the antibacterial activity of CEO. Research has demonstrated that bioactive phytochemicals like eugenol and cinnamaldehyde are responsible for the antibacterial properties of CEO (El Atki et al., 2020; Błaszczyk et al., 2021). It has been verified that CEO exhibits broad-spectrum efficacy against a variety of gram-positive and gram-negative bacteria (Chuesiang et al., 2019). On the other hand, it was noted that the marketed product of MP (Bactroban^®^) exhibited no antibacterial influence against gram-negative *E-coli*; however, it showed a substantial effect against Staph. The reason behind that was that MP was ineffective against gram-negative bacteria because it cannot get through their outer membranes; however, it exhibited high efficiency against the majority of gram-positive bacteria (Savage and Milner, 2024). These findings conclude that the combination of MP and CEO in a single formula may have contributed to the increased antibacterial activity shown by the MP–NLC-gel, according to the obtained data. Additionally, it was reported that antimicrobial agents that have been encapsulated into NLC enhanced the disposition of the antibacterial drugs, which could increase the effectiveness of the medications (Khan et al., 2023).

**TABLE 6:** Antibacterial activity of studied formulations against unlike bacterial strains.

Bacterial type	Inhibition zone (cm)		
	MP–NLC-gel	Marketed (Bactroban^®^)	Blank-NLC-gel
*Staphylococcus aureus*	4.3 ± 0.15 *	3.8 ± 0.13 *#	3.51 ± 0.11
*E. coli*	2.5 ± 0.10 #	Negative	2.3 ± 0.16 #

Values are NLC-gel-expressed as mean ± SD. * (*P* < 0.05) compared to blank formulation; and # (*P* < 0.05) compared to Bactroban^®^.

## 4 Conclusion

In the present study, central composite design was employed to develop and optimize the mupirocin-nanostructured lipid carrier. The optimum formula showed suitable particle size and high entrapment efficiency. For more convenient application of the formulation over the skin, the optimized formula was incorporated into a pre-prepared gel formula to provide mupirocin–NLC-gel. The gel formula demonstrated acceptable properties to be suitable for topical application. Most importantly, the formulated NLC-gel showed good physical stability, following 6 months of storage at different conditions. Additionally, the antibacterial activity of mupirocin in the NLC-gel formulation was potentiated, which is supposed to be due to the presence of cinnamon oil. Conclusively, the nanostructured lipid carrier could be considered an alternative nanolipid carrier anticipated for topical application. It is highly recommended to carry out definite future research studies such as *in vivo* experiments in order to evaluate the safety and clinical efficacy of NLC formulation.

## Data Availability

The original contributions presented in the study are included in the article/supplementary material; further inquiries can be directed to the corresponding authors.
